# Metabolomic and lipidomic plasma profile changes in human participants ascending to Everest Base Camp

**DOI:** 10.1038/s41598-019-38832-z

**Published:** 2019-02-19

**Authors:** Katie A. O’Brien, R. Andrew Atkinson, Larissa Richardson, Albert Koulman, Andrew J. Murray, Stephen D. R. Harridge, Daniel S. Martin, Denny Z. H. Levett, Kay Mitchell, Monty G. Mythen, Hugh E. Montgomery, Michael P. W. Grocott, Julian L. Griffin, Lindsay M. Edwards

**Affiliations:** 1 Centre for Human and Applied Physiological Sciences, King’s College London, London, UK; 2 Department of Physiology, Development and Neuroscience, University of Cambridge, Downing Street, Cambridge, UK; 3 Centre for Biomolecular Spectroscopy and Randall Division of Cell and Molecular Biophysics King’s College London Guy’s Campus London, London, UK; 4 NIHR BRC Nutritional Biomarker Laboratory, University of Cambridge, Pathology building level 4, Addenbrooke’s Hospital, Cambridge, UK; 5 University College London Centre for Altitude Space and Extreme Environment Medicine, UCLH NIHR Biomedical Research Centre, Institute of Sport and Exercise Health, First Floor, 170 Tottenham Court Road, London, W1T 7HA UK; 6 Critical Care Unit, Royal Free Hospital, Pond Street, London, NW3 2QG UK; 7 Southampton NIHR Biomedical Research Centre, University Hospital Southampton, Southampton, UK; 8 Integrative Physiological and Critical Illness Group, Division of Clinical and Experimental Science, Faculty of Medicine, University of Southampton, Southampton, UK; 9 University College London Hospitals National Institute of Health Research Biomedical Research Centre, London, UK; 10 Centre for Human Health and Performance, Department of Medicine, University College London, London, UK; 11 Department of Biochemistry and Cambridge Systems Biology Centre, University of Cambridge, Tennis Court Road, Cambridge, UK; 12 Respiratory Data Sciences Group, Respiratory TAU, GlaxoSmithKline Medicines Research, Stevenage, UK

## Abstract

At high altitude oxygen delivery to the tissues is impaired leading to oxygen insufficiency (hypoxia). Acclimatisation requires adjustment to tissue metabolism, the details of which remain incompletely understood. Here, metabolic responses to progressive environmental hypoxia were assessed through metabolomic and lipidomic profiling of human plasma taken from 198 human participants before and during an ascent to Everest Base Camp (5,300 m). Aqueous and lipid fractions of plasma were separated and analysed using proton (^1^H)-nuclear magnetic resonance spectroscopy and direct infusion mass spectrometry, respectively. Bayesian robust hierarchical regression revealed decreasing isoleucine with ascent alongside increasing lactate and decreasing glucose, which may point towards increased glycolytic rate. Changes in the lipid profile with ascent included a decrease in triglycerides (48–50 carbons) associated with *de novo* lipogenesis, alongside increases in circulating levels of the most abundant free fatty acids (palmitic, linoleic and oleic acids). Together, this may be indicative of fat store mobilisation. This study provides the first broad metabolomic account of progressive exposure to environmental hypobaric hypoxia in healthy humans. Decreased isoleucine is of particular interest as a potential contributor to muscle catabolism observed with exposure to hypoxia at altitude. Substantial changes in lipid metabolism may represent important metabolic responses to sub-acute exposure to environmental hypoxia.

## Introduction

Reduced cellular oxygen availability (hypoxia) is a feature of many disease states. Oxygen delivery may be globally impaired by diseases of the heart or lung, or by anaemia (in which the concentration of oxygen-carrying haemoglobin is reduced); or regionally or locally impaired by macrovascular and microvascular disease respectively^1,2^. Diffusion of oxygen in the lungs and/or tissues may be impaired through accumulation of parenchymal fluid and/or inflammatory changes^1^. Meanwhile, ascent to high altitude resulting in exposure to environmental hypobaric hypoxia may also reduce tissue oxygen availability: the associated fall in barometric pressure is in turn associated with a decrease in the inspired partial pressure of oxygen (PO_2_). Increasing numbers of lowlanders travel to high altitude destinations, whilst ~7% of the world’s population (440 million people) reside above 1500 m^3^).

Adjustment to hypoxic conditions (acclimatisation) involves responses that mitigate reduced oxygen delivery, including an increase in minute ventilation and erythropoiesis^4^. Acclimatisation also requires a metabolic response to help match ATP synthesis and demand in the face of decreased oxidative capacity and increased oxidative stress^5–7^.

To date, studies examining metabolic acclimatisation in healthy human lowlanders have predominantly focused upon tissue specific responses, particularly those of skeletal muscle. These suggest a shift away from oxidative processes including β-oxidation, TCA cycle activity and oxidative phosphorylation (reviewed in^5^) and towards increased reliance upon carbohydrate metabolism. Enhanced glycolytic capacity has been suggested by increased intramuscular levels of glycolytic intermediates^8^ and hypoxic inducible factor 1-α (HIF-1α) mediated upregulation of glucose transporters and glycolytic enzymes^9,10^.

Hypoxia-induced alterations to lipid storage and mobilisation include a fall in circulating high density lipoproteins alongside increased triglyceride (TG) concentrations^11^, inhibition of lipoprotein lipase activity^12^ and suppression of *de novo* lipogenesis^13,14^. These responses are likely to be mediated at the transcriptional level through HIF-1/2α^15–17^ and may be affected by changes in circulating catecholamines^6,11^, which are known to stimulate lipolysis via hormone sensitive lipase^18^. In addition, a transcriptional regulator of fatty acid oxidation in the liver, heart and muscle, peroxisome proliferator activated receptor α (PPARα), has been identified as a key regulator of hypoxic metabolic remodeling processes, with the metabolic adaptations of native high altitude Sherpa populations being linked to a putatively advantageous allele for the PPARα gene^8,19,20^.

Whilst details on metabolic acclimatisation to hypoxia are emerging, there are profound differences between studies in the duration and degree of hypoxic exposure^5^, which tends to be applied to highly selected, small groups of participants (e.g.^21,22^). Amongst those involving larger cohorts, there has been a failure to use standardised ascent profiles^23–25^. Further, many studies have been tissue-specific, with little attention paid to circulating features indicative of global systemic metabolic responses.

Metabolomic and lipidomic analyses of biofluids measure a large number of variables of interest within the metabolic and lipid systems, thus providing a sensitive measure of functional biological phenotype (reviewed in^26^). The power of such analyses has been demonstrated in the identification of biomarkers related to the diagnosis or prognosis of a range of diseases^27^. The application of such methodology to the examination of responses to human hypobaric hypoxia has the potential to elucidate a metabolic signature of altitude exposure^28,29^. The few studies that have adopted this approach in the context of high altitude exposure have again employed small, select study cohorts^30,31^.

In order to better describe the human response to hypobaric hypoxia, we thus performed a large scale prospective metabolomic and lipidomic analysis of plasma samples taken from 198 participants across 5 timepoints upon their ascent to Everest Base Camp (EBC, 5,300 m) as part of the Caudwell Xtreme Everest expedition^32^.

## Methods

The study design, risk management plan and individual protocols for the Caudwell Xtreme Everest (CXE) Expedition were approved by the University College London (UCL) Research Ethics Committee (in accordance with the declaration of Helsinki). All methods were performed in accordance to relevant guidelines and regulations. The study was designed and conducted by the UCL Centre for Altitude Space and Extreme Environment Medicine (CASE). Both verbal and written informed consent was obtained from all participants.

### Participants

Details on subject recruitment and characteristics have been outlined previously^32^. Briefly, participants included males and females aged over 18 with no upper age limit, who were required to pass two separate health screening stages^32^. From this screening process, 198 healthy volunteers participated in the study.

### Study design

Ascent profiles, details on logistics as well as barometric pressure (P_B_), inspired partial pressure of oxygen (P_i_O_2_) and temperature at the respective altitude laboratories have been reported previously^32^. Baseline measurements were performed in London (LDN) in a laboratory at UCL (75 m above sea level, PiO_2_ 19.7 KPa) between January 4^th^ and February 26^th^ 2007. Field studies were performed between 31^st^ March and 6^th^ June 2007 at laboratories set up at the following locations (altitudes expressed as meters, m): Kathmandu (KTM, 1,300 m, PiO_2_ 16.8 KPa), Namche (NAM, 3,500 m, PiO_2_ 12.7 KPa), Pheriche (PHE, 4,250 m, PiO_2_ 11.6 KPa) and Everest Base Camp (EBC, 5,300 m, PiO_2_ 9.9 KPa). Data collection, including plasma sample collection, was performed at each of these time points.

All participants underwent an identical ascent profile, arriving at EBC on day 11. To maintain an identical pattern of hypoxic exposure, participants’ movements were restricted on rest days, such that no subject ascended or descended more than 300 vertical meters from the laboratory altitude. The total trekking distance achieved over 11 days from Lukla airport to EBC was 50.7 km, with 5 days of rest over this course. The distance covered per day, taken at a gentle pace over the course of the day, was thus as follows: Lukla to NAM 9.1 km/day, NAM to PHE 10.3 km/day, PHE to EBC 5.9 km/day.

### Physiological variables

Body weight and arterial O_2_ saturation (SpO_2_), were determined prior to any oral intake on the morning that blood samples were obtained^6^.

### Plasma Sample Analysis

#### Blood sampling

All blood samples were taken on a rest day the morning after arrival at each altitude location, being a minimum of 16 hours post exercise. Subjects were in a rested, fasting state and blood was taken from the antecubital vein and collected in 10 ml BD ethylenediamine-tetra-acetic acid (EDTA) blood tubes (Southern Syringe Services LTD). Plasma was separated from blood cells by centrifugation of whole blood at 800 *g* for 15 min and immediately frozen in 1 ml aliquots in liquid nitrogen. Samples were kept below −80 °C until analysis.

#### Plasma preparation

Plasma samples were defrosted at room temperature. A 1:1:1 extraction was performed^33^, with 500 µl of methanol (Hypergrade for LC-MS, Merck) and 500 µl of chloroform (HiPerSolv Chromanorm for HPLC, VWR international), both ice cold, being added to 500 µl of plasma in a 1.5 ml Eppendorf. This was vortexed for 2 min, left to stand at −20 °C for 30 min, and then centrifuged for 3 min at 9100 *g* (Force 1624 Microcentrifuge) to yield upper (methanol/aqueous, fraction) and lower phases (chloroform/lipid, fraction). 700 µl of the upper phase and ~200 µl of the lower phase were pipetted into Eppendorf tubes for nuclear magnetic resonance spectroscopy (NMR) and mass spectrometry analysis, respectively.

#### Upper phase/methanol fraction

The upper fraction was dried down at 30 °C for 4 hours using a vacuum centrifuge (Eppendorf Concentrator 5301) and then re-suspended in 600 µl of the following NMR buffer: double distilled H_2_O containing 5% D_2_O for NMR (Acros organics, CAS: 7789-20-0) and 1 mM 3-trimethylsilyl-1-propanesulfonic acid sodium salt (DSS) (Aldrich, 178837-5 G). D_2_O was required for the magnetic lock frequency and DSS was used as a chemical shift reference. The resulting solution was transferred to 5 mm NMR tubes within a 96-tube rack.

#### Proton (^1^H-) NMR spectral acquisition and processing

^1^H-NMR spectra of plasma samples were obtained using a Bruker Avance III 700 MHz spectrometer (Bruker Biospin, Karlsruhe, Germany) as described previously^34^. All samples were analysed on a participant by participant basis, meaning that plasma taken from the same participant at all 5 time points was defrosted and treated at the same time and run within the same NMR experiment. This was to ensure any daily differences in experimental procedure were removed from analysis when data between time points were compared.

Resulting spectra were processed using TopSpin (Bruker, Karlsruhe, Germany). Spectra were converted from time to frequency domain using a Fourier transform. The phase was adjusted and spectra aligned so that the DSS peak corresponded to 0 ppm through use of the *i*coshift program in Matlab^35^. Due to the degree of noise present, the aromatic region was excluded and only the aliphatic spectral region analysed. Normalisation and scaling of this spectral region were performed through use of Probabilistic Quotient normalisation^36^ and Pareto scaling^37^, respectively (Supplementary Fig. 1).

Initial principal components analysis (PCA) on this region revealed a separation into two regions that was not due to variation dependent on the experiment i.e. a change with location and thus with altitude (Supplementary Fig. 2). Instead, it was likely due to sample acquisition differences such as slight alterations in gain between NMR batch runs, as the samples were run in two batches at different times. A bias trend was identified to capture 77% of this variance (Supplementary Fig. 3) and was subsequently normalised using EigenMS (Supplementary Fig. 4)^38–40^. As all samples from any single subject were run at the same time, they were contained within one of the two batch effect groups.

Following this, the full resolution spectra were binned at a ratio of 10:1. This ratio was found to sufficiently reduce the size of the data set, whilst retaining maximal information. The total number of bins was 1203. Putative identification of the metabolites associated with the peaks undergoing significant changes was undertaken using Chenomx software (Chenomx NMR Suite 7.1).

#### Lower phase/lipid fraction analysis by direct infusion mass spectrometry (DIMS)

All solvents used were of LC-MS grade or better and were ordered from Sigma Aldrich (Gillingham, UK). Quality controls (QC’s) were derived from pooling all samples and serially diluting with chloroform. Internal standards for key lipid species or blanks (either PBS or chloroform) were prepared to run alongside the sample lipid fraction. All internal standards were obtained from Avanti Polar Lipids (Alabaster, AL, USA) with the exception of undecanoic acid and trilaurin (Sigma Aldrich).

The sample, QC’s and blanks (30 µl) were placed in a pre-defined random order across 96-well plates (Plate+, Esslab, Hadleigh, UK). To this, 750 µl methyl tert-butyl ether (MTBE) was added, along with 150 µl of internal standard mix, containing the following six internal standards: 1,2-di-o-octadecyl-sn-glycero-3-phosphocholine (0.6 µM), 1,2-di-O-phytanyl-sn-glycero-3-phosphoethanolamine (1.2 µM), C8-ceramide (0.6 µM), N-heptadecanoyl-D-erythro-sphingosylphosporylcholine (0.6 µM), undecanoic acid (0.6 µM), and trilaurin (0.6 µM). The blanks remained as such through addition of 150 µl chloroform instead of internal standard. The plate was subsequently shaken for 30 s.

Using a VIAFLO 96/384 electronic pipette (Integra), 25 µl of the resulting sample mixture was transferred to a glass coated 384 well plate and 90 µl mass spectrometry (MS) mix [7.5 mM NH_4_Ac IPA:MeOH (2:1)] added. The plates were then sealed using Corning aluminium micro-plate sealing tape (Sigma Aldrich Company, UK) and kept at −80 °C until required for DIMS analysis.

Lipidomics was performed as described previously^41^ using chip-based nanospray with an Advion TriVersa Nanomate (Advion, Ithaca, USA) interfaced to the Thermo Exactive Orbitrap (Thermo Scientific, Hemel Hampstead, UK). Briefly, a mass acquisition window from 200 to 2000 m/z and acquisition in positive and negative modes were used with a voltage of 1.2 kV in positive mode and −1.5 kV in negative mode and an acquisition time of 72 s.

Acquired spectral raw data were processed as described previously^41^ using an in-house bioinformatics platform based on XCMS^42^. With the use of predefined rejection lists and mass defect filters, this performed sample-specific mass re-calibration using predefined sets of internal standards and the removal of commonly present contaminant ions (often associated with plasticizers). The raw data were converted to.mzXML (using MSconvert^43^ with peakpick level 1), parsed with R and 50 (scan from 20 to 70) spectra were averaged per sample using XCMS^42^, with a signal cutoff at 2000. The files were aligned using the XCMS^42,44^ grouping function using “mzClust” with a m/z-window of 22 ppm and a minimum coverage of 60%. Automated compound annotation was carried out using both an exact mass search in compound libraries as well as applying the referenced Kendrick mass defect approach. Signal normalisation was performed by summing the intensities of all detected metabolites to a fixed value to produce a correction factor for the efficiency of ionisation.

As described previously^45^, exact masses were fitted to the lipid maps library and subsequently annotated to the peak. This converts initially roughly 4000 features (spectral regions associated with an analyte). These features were then considered within the parameters of our model, leading to 9 identified lipid species excluding isotopes.

### Data modelling using Bayesian Statistical Methods

#### Model justification

The data recorded in the present study were naturally hierarchical: there were multiple measurements of each metabolite in each subject; yet for the present experiment, the aim was to identify those metabolites that were consistently affected by altitude across the entire study population. For this reason, we adopted a hierarchical approach to statistical modelling – specifically we fitted a hierarchical Bayesian model for each metabolite. This model comprised robust regression models (robust in that the residuals were modelled with a *t*-distribution^46^) for each binned ^1^H-NMR region or lipid variable at the participant level; the parameters from these regression analyses (specifically the slope and intercept, β1 and β0) were themselves modelled with normal distributions. Beyond the more general contrasts between using Bayesian over frequentist statistics (a recent discussion of which can be found here^47^), several advantages are specific to this application. First, a full distribution of credible regression lines for each metabolite in each subject was generated. Second, by fitting the complete hierarchical model in a single step, and estimating all distributions simultaneously, the estimates of the regression lines for each individual subject are constrained by the overarching distribution of the lines for that metabolite, a phenomenon known as shrinkage^48,49^. Hence slopes that are very unlikely for an individual subject (when the slopes for the remainder of the group are considered) are excluded unless compelling evidence exists in the data.

Despite the advantages, Bayesian statistics are computationally expensive. Therefore, simple frequentist hierarchical models were fitted initially to all variables in order to identify those variables likely to be of interest for further modelling (in other words, those metabolites whose mean slopes were not zero). Only those metabolites whose mean slopes (from simple hierarchical modelling) were >±1.96 SDs from the mean were carried forward for full Bayesian modelling. These slopes were later compared to those derived using Bayesian methods and found to be reasonable estimates (Supplementary Fig. 5). Finally, Bayesian inference is somewhat different to frequentist approaches (e.g. there are no p-values). Hence, we adopted the criterion that metabolites should not include 0 in the high-density (95%) interval of the posterior distribution of z-transformed slopes. Together, this means that two levels of filtering were employed to ensure that only those slopes undergoing large degrees of change were considered.

#### Model details

The full Bayesian hierarchical model used here was described previously^50^. A model was fitted for every metabolite of interest. Data were *z*-transformed before fitting to reduce correlation between slopes and intercepts (which presents difficulties for the MCMC algorithm used in JAGS). Each model comprised linear sub-models of the standard form fitted to the normalised metabolite measurements for each subject, using a *t*-distribution for the residuals. The distribution of parameters for the *t*-distribution (σ and ν, the normality parameter) were estimated once per metabolite, not for each subject. The slopes and intercepts from each of these models were themselves modelled using normal distributions with parameters μ_0_ (the mean intercept), σ_0_ (the standard deviation of the intercepts), μ_1_ (the mean slope) and σ_1_ (the standard deviation of the slopes). Hence for each metabolite, the modelling process generated distributions for σ, v, μ_0_, σ_0_, μ_1_ and σ_1_ and – for each subject – distributions for β_1_ and β_0_. Our primary interest was in the distributions of μ_1_. Non-informative priors were used for all parameters. Remembering that all data were *z*-transformed prior to analysis, all σ were given a broad prior uniform distribution (with parameters L = 10^−3^ and H = 10^3^ denoting the low and high limits), and ν a broad exponential distribution with a minimum of 1 and mean of 30 (at which point the distribution is approximately normal). For each normal distribution, μ was given a normal prior with parameters M = 0 and S = 10. Both the individual and group regression were assessed simultaneously and the individual regression lines constrained by the average regression lines.

Bayesian analysis was conducted using R and JAGS (Just Another Gibbs Sampler^51^ as described in Kruschke^50^). All code is available in supplementary materials.

Identified metabolites were presented with corresponding identification codes from the human metabolome database (HMDB). In the case of certain lipids where HMDB identification was not available, chemical entities of biological interest (ChEBI) were included.

### Calculation of Absolute Changes

For the aqueous phase metabolites, the binned ^1^H-NMR spectral regions corresponding to an identified metabolite were assessed. The change in spectral intensity between LDN and EBC was calculated and from this the median % change derived. Given that this was a measure of spectral intensity, no absolute value such as concentration/ml plasma could be given for the change, instead arbitrary units were employed. The LDN and EBC values were also corrected to the LDN value, to give a normalised ratio of change. The same approaches were employed for assessment of the degree of change in lipid abundance, although this only included those definitively annotated lipids.

### Statistical analysis

Analysis was performed in GraphPad Prism 7. The LDN and EBC values for SpO_2_ and body weight data were abnormally distributed, as assessed using the D’Agostino-Pearson normality test. Therefore, a Wilcoxon matched-pairs signed rank test was employed for analysis.

The Δ abundance of metabolites/lipids from LDN to EBC that were identified as undergoing large changes with ascent were plotted against the Δ SpO_2_ or body weight from LDN to EBC. For the aqueous fraction metabolites, this specifically refers to the Δ of spectral region intensity undergoing the greatest change, and for lipids, the definitively identified lipid variables. Data that followed a Gaussian distribution were tested using a Pearson Correlation Coefficient. A non-parametric Spearman rank correlation (two tailed) was applied for non-normally distributed data. If a significant relationship was identified, linear regression analysis was subsequently performed.

## Results

### Subject Characteristics, Body Weights and Arterial O_2_ Saturations

One hundred and ninety-eight participants (125 male, 73 female, 44 ± 14 (mean, ± SD) years of age, BMI 25.1 ± 3.2 kg/m^2^) participated in the expedition to EBC^32^.

**Figure 1:**
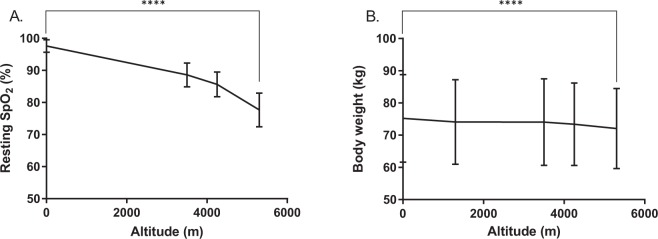
Changes in physiological variables with ascent to EBC. This includes recording of resting arterial O_2_ saturation (SpO_2_) (**A**) and body weight (kg) (**B**) at each time point upon ascent, with the midpoint representing the mean, ± SD. Data was tested using a Wilcoxon matched-pairs signed rank test between LDN to EBC, ****p < 0.0001, n = 146–188.

The degree of hypoxic exposure experienced at altitude was reflected through progressive decreases in resting SpO_2_ with ascent. Overall, a 20.4% median decrease in SpO_2_ (n = 187, p < 0.0001) from 98% at LDN to 78% at EBC was observed (Fig. 1A). Body weight also progressively fell by a median of 3.7% (3 kg) between LDN and EBC (n = 157, p < 0.0001,) (Fig. 1B).

### Plasma Metabolomic/Lipidomic Analysis

A total of 965 samples were obtained for metabolomic analysis on the aqueous fraction using NMR, and lipidomic analysis on the lipid fraction using DIMS. Between 182 and 198 samples were analysed from each altitude location, totaling up to 5 samples per volunteer (1 per altitude location). The reason for differences in sample number was due to participants failing to arrive at the laboratory^32^ or insufficient amounts of plasma being obtained for subsequent analysis. Bayesian robust hierarchical regression was used to identify metabolites that were robustly related to changes in altitude, from both the NMR and MS data. In particular, the focus was on those metabolites where the 95% high-density interval (a similar concept to 95% confidence intervals in traditional statistics) of estimates of the overall slope of metabolite abundance vs. altitude did not contain 0.

#### Plasma aqueous fraction analysis

**Figure 2:**
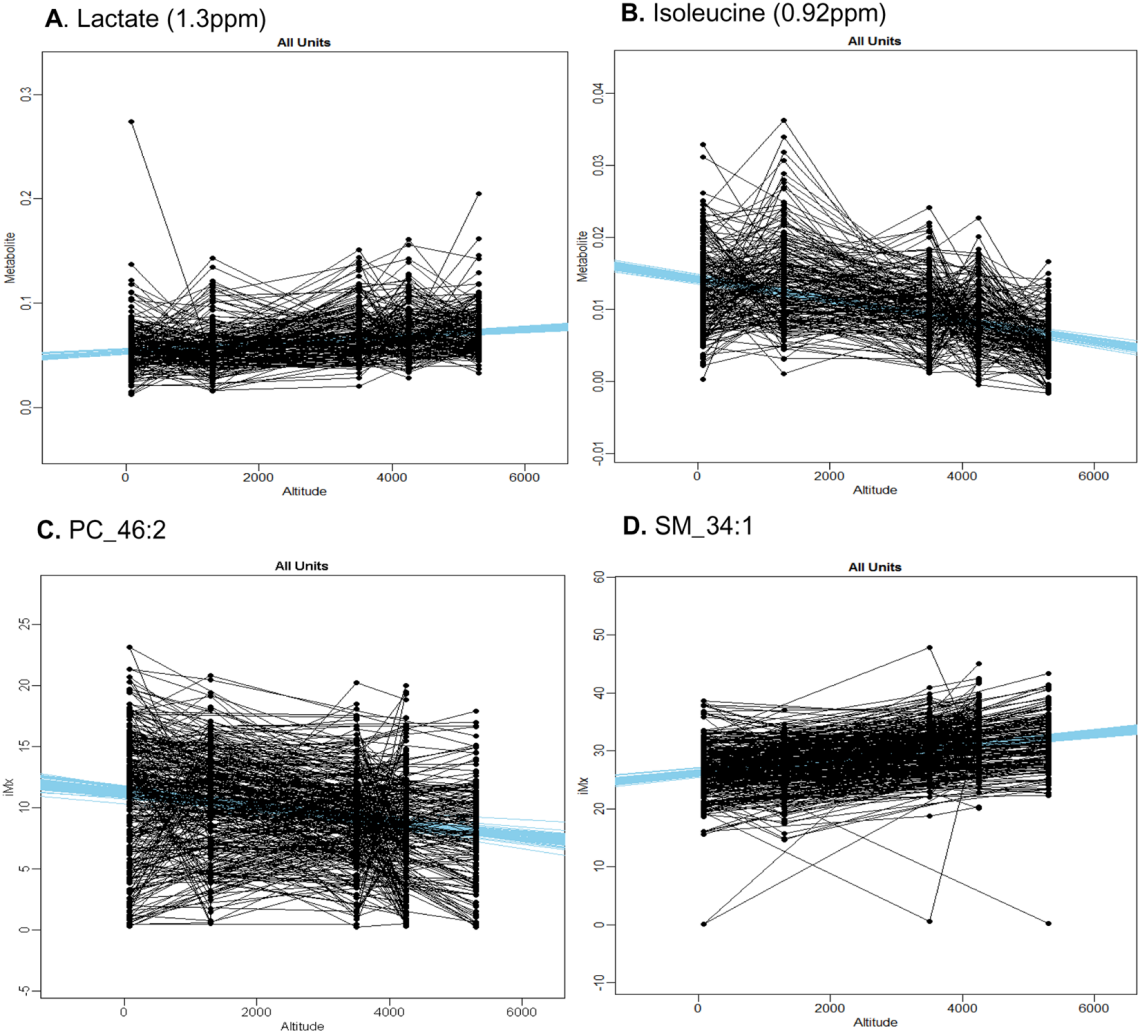
An example of the full subject group response of aqueous metabolites or lipids that demonstrate a significant trend with increasing altitude, identified using Bayesian hierarchical modelling. Example plots of lactate (**A**) and isoleucine (**B**), with corresponding ^1^H-NMR regions, phosphocholine (PC) 46 carbons: 2 double bonds (46:2) (**C**) identified in negative ion mode and sphingomyelin (SM) 34:1 (**D**) identified in positive mode. Representative regression lines in blue have been drawn from the fitted distribution. This group distribution (all units) was informed from the most likely distribution at the level of the individual. Y axes metabolite units are arbitrary units, derived from the spectral intensity changing per km altitude.

**Figure 3:**
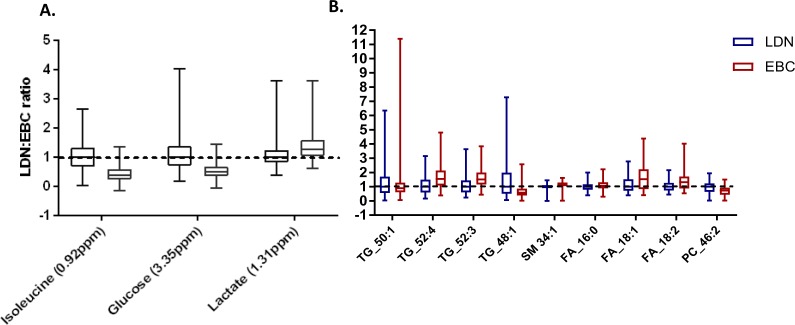
Alterations in aqueous metabolite and lipid abundance from London (LDN) to Everest base camp (EBC). Aqueous metabolite ^1^H-NMR spectral regions (**A**) and lipids (assessed using DIMS) (**B**) undergoing the largest degree of change with ascent to EBC, identified using Hierarchical Bayesian statistics. Values are corrected to LDN, and so are expressed as a ratio of LDN: EBC, with a value of 1 indicative of no change. Presented as minimum to maximum box and whisker plots, with the middle line representing the median and the box the interquartile range (25^th^ to 75^th^ percentiles).

An example of the Bayesian model output for the overall group response is displayed in Fig. 2A,B, with examples of the individual response being displayed in Supplementary Fig. 6. A summary of aqueous metabolite changes are outlined in Table 1, with differences in peak intensity from LDN to EBC, expressed as a ratio, presented in Fig. 3A. The changes include a progressive decrease in glucose (D-Glucose HMDB00122), an increase in lactate (L-Lactic acid, HMDB00190) and a decrease in the branched-chain essential amino acid isoleucine (L-Isoleucine HMDB00172) with ascent to EBC. From the spectral regions corresponding to these metabolites, specific spectral peaks undergoing the largest degree of change were identified as: 1.31ppm for lactate (27.4% increase), 3.35ppm for glucose (52.4% decrease) and 0.92 ppm for isoleucine (60.5% decrease).

**Table 1:** Spectral intensity, % change and credible regression slope change of the ^1^H-NMR spectral regions identified as undergoing the largest degree of change with increasing altitude.

Metabolite	Binned spectral region (ppm)	LDN intensity (AU)	EBC intensity (AU)	Δ% LDN to EBC	Slope (AU/km)	Increasing or decreasing with altitude
Isoleucine	0.92	0.012	0.0049	−60.5	−0.00142	Decreasing
Glucose	3.35	0.016	0.0081	−52.4	−0.0014	Decreasing
Lactate	1.31	0.057	0.0719	27.4	0.00361	Increasing

#### Plasma lipid fraction analysis

Two examples of the output from Bayesian hierarchical modelling of lipidomic data of the full participant group are presented in Fig. 2C,D. All lipid species identified as undergoing large changes with ascent (defined in the same way as for NMR spectral regions, see above) are summarised in Table 2, with the LDN:EBC ratio presented in Fig. 3B. The largest % lipid increase was identified as triglyceride (TG) with carbon chain: double bond ratios of 52:3, with a median increase from LDN to EBC of 53.9%. This occurred alongside an increased TG 52:4 (CHEBI: 84660) and in the most abundant non-esterified fatty acids within adipose tissue. This includes the saturated palmitic acid (16:0) (HMDB00220) and unsaturated linoleic (18:2) (HMDB00673) and oleic (18:1) (HMDB00207) acids. An increase was also observed in sphingomyelin (SM) 34:2 (CHEBI:64587). The largest lipid % decrease was that of TG 48:1 (CHEBI: 85726), with a median decrease of 43.1%. This occurred alongside decreased TG 50:1 (50:1 (CHEBI: 84665) as well as phosphatidylcholine (PC) 46:2 (CHEBI: 72430).

**Table 2:** Abundance, % change and credible regression slope change for lipid variables identified as undergoing the largest degrees of change with increasing altitude.

	Carbon chain length: double bond	Mode of ion detection	LDN abundance (AU)	EBC abundance (AU)	Δ% LDN to EBC	Δ Slope (AU/km)
***Lipids increasing with ascent***						
Triglyceride	52:3	Positive	30.1	45.0	53.9	1.59
Triglyceride	52:4	Positive	10.72	16.50	53.7	0.529
Oleic acid	18:1	Negative	36.0	54.6	27.1	2.349
Linoleic acid	18:2	Negative	17.83	23.39	23.7	0.828
Sphingomyelin	34:1	Positive	26.8	30.8	15.7	0.001
Palmitic acid	16:0	Negative	110.4	116.7	12.4	2.176
***Lipids decreasing with ascent***						
Triglyceride	48:1	Positive	4.25	2.42	−43.1	−0.334
Phosphatidylcholine	46:2	Negative	11.8	8.7	−25.0	−0.597
Triglyceride	50:1	Positive	10.95	9.69	−8.43	−0.524

### Correlation analysis

To assess whether a relationship existed between altitude dependent changes in metabolites/lipids from LDN to EBC and the specific physiological variables presented here, correlation plots were constructed.

**Figure 4:**
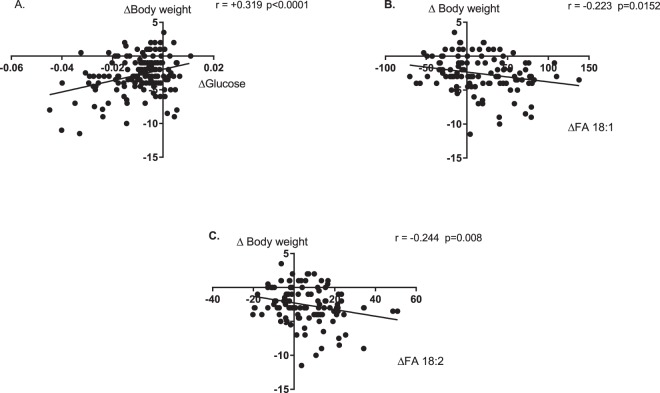
Correlation of Δ glucose or fatty acids (FA) vs. Δ body weight. Glucose (**A**), unsaturated FA 18:1 (**B**) and 18:2 (**C**), shown as carbon: double bond ratio. Δ calculated as EBC-LDN. Correlation analysis performed using Pearson rank correlation coefficient. Significant (p < 0.01) correlations analysed further using linear regression.

Plots of changes in aqueous metabolites against body weight revealed a significant correlation between changes in glucose and body weight (p = 0.007), with decreased body weight being associated with decreased plasma glucose (Fig. 4A). Plots of the change in fatty acid vs. body weight from LDN to EBC revealed a significant correlation between the unsaturated oleic (18:1) (p = 0.0127) and linoleic (18:2) (p = 0.0062) FA’s and body weight from LDN to EBC, with loss of body weight at altitude being associated with increasing levels of oleic and linoleic acids (Fig. 4B,C). No significant correlations were observed for changes in the other identified aqueous metabolites or lipids and body weight. Equally, no significance was observed between changes in any metabolite or lipid and SpO_2_.

## Discussion

With exposure to reduced PiO_2_ during ascent to EBC, SpO_2_ fell proportionally by ~20%. We examined the metabolic response to this hypoxic exposure. We made efforts to distinguish this from responses to the inevitable potential confounders of an expedition of this nature, such as dietary changes. To this end, we used robust statistical methods to identify metabolites that changed with altitude and hierarchical methods that captured the full structure of the data. That these findings are consistent with known biology serves to validate the methods used.

Specific changes in aqueous metabolites include a decrease in the glycolytic substrate glucose alongside an increase in the glycolytic product lactate. This pattern of change has long been associated with increased glycolytic rate in the context of human cancer cell lines (e.g.^52^). In the context of high altitude exposure, the changes observed support prior observations of decreasing blood glucose concentrations, increasing muscle glycolytic intermediates and increasing blood lactate levels in lowlanders ascending to high altitude^6,8^. They are also in keeping with prior human, animal and cellular studies showing increased translocation of glucose transporters (GLUT-1 and GLUT-4) to the plasma membrane,^53,54^, upregulation of lactate dehydrogenase^22,55^ and a shunting of pyruvate away from entry into the TCA cycle and towards lactate formation^9^, all attributed to increased HIF-1α activity. Together, this therefore suggests the changes observed are a result of increased reliance upon anaerobic glycolysis at rest following ascent to high altitude. However, it is also worth noting that a decrease in plasma glucose may result from increased insulin secretion, which has been shown previously to occur upon altitude exposure^6^.

Lipidomic analysis revealed alterations to the main constituent of body fat, TGs. Specifically, circulating levels of TGs 52:4 and 52:3 increased whereas TGs 50:1 and 48:1 decreased. Those TGs containing 48–50 carbons are typically associated with *de novo* lipogenesis a process by which excess carbohydrates are converted to fatty acids and subsequently to TGs for storage^45,56^. This association has been demonstrated in human intervention studies^45^. The fall in concentrations of TGs 48:1 and 50:1 with ascent to EBC may thus be linked with suppressed *de novo* lipogenesis, an effect known to be mediated by HIF-1α^13,14^. The increase in TGs 52:4 and 52:3 are likely a compensatory change to retain a balance in circulating TGs. However, further analysis directly measuring *de novo* lipogenesis would be required to come to a firm conclusion regarding these changes.

The combination of decreased TGs alongside increased circulating levels of the most abundant fatty acids (including palmitic, linoleic and oleic acids), may be indicative of fat store mobilisation. This process can be induced via activation of the sympathetic nervous system (catecholamines activate cAMP-dependent phosphorylation of hormone sensitive lipase, which stimulates TG mobilisation^57^). Sympathetic activation is known to occur in response to hypobaric hypoxia, with an increase in efferent activity directed towards the heart, kidneys, vasculature and skeletal muscle^58,59^. Indeed, alterations in adrenaline/noradrenaline have been reported in previous investigations of the CXE expedition team, peaking at Namche Bazaar (3,500 m)^6^. Together this indicates that fat stores may become mobilised through hypoxic stimulated sympathetic activity, although again, further work would be required before a firm conclusion can be drawn.

Catecholamine stimulated breakdown of TG’s to release fatty acids can be impaired through the action of insulin. Increased circulating levels of fatty acids (notably palmitic, oleic and linoleic acids) are associated with insulin resistance^60–62^, including in models of obesity through a mechanism reliant upon c-Jun amino terminal kinases (JNKs)^63^. Indeed, in a linked study by our group, participants remaining at EBC or above for prolonged periods exhibited a substantial increase in insulin and C-peptide^6^. It is therefore possible that the mobilisation of fat stores indicated in the present study, resulting in an increase in circulating fatty acids, is a causative factor for the spike in insulin concentrations observed in response to longer term exposure^6^.

The rise in circulating fatty acid levels may also contribute to previous reports of impaired β-oxidation capacity at high altitude^5^. Indeed, skeletal muscle mitochondrial respirometry carried out in a different cohort of lowlander participants undertaking similar ascent to that reported here, revealed impairment of skeletal muscle fatty acid oxidative phosphorylation at EBC along with an intramuscular accumulation of fatty acid intermediates^8^.

Lipidomic analysis also revealed changes in key membrane components, including a decrease in PC 46:2. PCs are key constituents in the construction of lipoproteins, in particular very low density lipoprotein (VLDL)^64^, which is required for lipid transportation from the liver to cells. This observation may thus suggest that hypobaric hypoxia could affect this process. Disrupted lipoprotein transport, with inhibition of lipoprotein lipase activity in adipose tissue and pre-adipocytes, has been reported in response to chronic intermittent^65^ and acute hypoxia^66^, respectively.

An entirely novel change identified in the aqueous metabolites was a decrease in the essential branched-chain amino acid (BCAA) isoleucine with ascent. This is in contrast to prior studies investigating plasma responses of rats exposed to acute hypoxia (9.5% O_2_, 5–18 hrs)^67^ and in HeLa cells (1% O_2_)^68^, which reported an increase in isoleucine levels. This disparity may relate to differences in hypoxic exposure, with much more prolonged and progressive hypoxic exposure being adopted in our study, and the use of human participants.

In addition to their essential role in building muscle tissue, BCAA’s are regulators of key cellular signaling pathways including that of the mammalian target of rapamycin (mTOR), a central regulator of cell metabolism, growth and survival^69^. The upregulation of mTOR signaling to induce protein synthesis is reliant upon an optimal ratio of BCAA’s^70^. In the present study, decreasing isoleucine levels were not matched by changes in the levels of leucine or valine to the same extent, as these amino acids did not meet the model criteria. Thus, whilst the consequences of the fall in circulating isoleucine in the context of hypoxic exposure remain to be determined, an impact upon muscle protein synthesis and potentially muscle catabolism, is likely. Indeed, mTOR protein levels have been reported to decrease in response to 7–9 days exposure to 4559 m in human vastus lateralis^71^.

In the present study body mass fell by an average of 3%, a finding in line with the commonly reported catabolic response to high altitude. First reported by Pugh in 1962^72^, a fall in body mass with altitude exposure has since been linked to loss of lean mass attributed to muscle catabolism^73,74^. Attenuation of hypoxia-induced loss in body mass has been reported with BCAA supplementation^75^, whereas supplementation solely with leucine was not effective^76^. Together with the results of the present study, this may therefore suggest that inclusion of isoleucine in dietary supplementation may be crucial if muscle catabolism is to be mitigated against.

To investigate whether metabolite changes were associated with either the degree of change in SpO_2_ or body weight with ascent, correlation analyses between metabolites/lipids and these factors were performed. This is in line with the notion that whilst changes in oxygen delivery are an essential part of hypoxic acclimatisation, alterations to metabolic processes modifying oxygen use at a cellular level are crucial^5,8^. Indeed, mechanisms altering oxygen delivery do not account for inter-individual performance at altitude^77,78^ and the hypoxic phenotypes of acute mountain sickness does not correlate well with degree of SpO_2_^79^. Correlation analysis did reveal that loss of body weight was associated with decreasing plasma glucose and increasing oleic and linoleic acids. It is not possible to determine whether this association may be caused by dietary alterations or loss of appetite. However, it is important to note that the loss of body weight was associated with increasing altitude exposure, which is in line with previous work outlining that appetite suppression at altitude is a hypoxic driven response mediated by changes in leptin signaling^80,81^. The present work thus highlights the importance of body mass changes with altitude in relation to metabolic shifts.

### Study limitations

In comparison to previous studies examining metabolic acclimatisation of human subjects to high altitude hypoxia both in the field and laboratory, this study is unique in its scale and design. To enable success, it required the adoption of a pragmatic approach, the nature of which precluded the addition of a control group. Whilst the insult of hypobaric hypoxia was undoubtedly severe at the altitude reached, it must be noted that the effects reported may have been influenced by other environmental factors, such as a change in temperature and UV exposure.

Other factors that are known to impact metabolic function that may have affected the metabolomic/lipidomic profiles are potential alterations to exercise and diet. Daily activity data was not recorded, however all subjects underwent identical ascent profiles for which the exercise burden each day was low in both intensity and duration, as demonstrated by trekking distance/day detailed in the methods. Given that the cohort of subjects were healthy and active people, habitually partaking in exercise as part of normal life thus makes acute changes in exercise unlikely. In addition, the timing of the blood draw (rest day morning, minimum 16 hrs post exercise in a fasted, rested state), again makes effects of an exercise insult upon resulting metabolite/lipid analysis unlikely.

Dietary changes are another potential confounding factor, given that food was consumed *ad libitum* and was limited to that available in Kathmandu and the remote trekking locations. Had dietary effects been present, they would most likely become apparent between London and Kathmandu, and following this Kathmandu to Namche. Initial analysis performed on the data sets using PLS-DA models demonstrated that the most accurate, robust separation in lipid positive mode was apparent between London and EBC rather than the London, Kathmandu and Namche comparisons (Supplementary Fig. 7). In addition, the focus of the Bayesian model output is the regression line, along which is plotted each altitude location. Whilst fluctuations are apparent between altitude locations, the credible regression lines are shown to be robust to relatively minor shifts. Together, this suggests the major factor in influencing the direction of the resulting trend is altitude. However, we would recommend that future studies examining metabolomic/lipdomic profiles should include a standardised dietary intake, as well as a daily recording of exercise.

Finally, limitations of our statistical approach must be noted. The benefits of the robust Bayesian modelling approach have been outlined in the methods section. The downside of this is the vast computational power required. A compromise was therefore required to practically obtain results in a reasonable time scale, resulting in the model parameters outlined in the methods section. In addition to restricting computational power requirements, this approach also helped to focus analysis upon the ‘big changers’ within the profile. The restrictions placed upon the selection of those metabolites taken forward for Bayesian modelling may have limited metabolite/lipid identification. This was particularly apparent in the aqueous metabolite results whereby only 3 metabolites were taken forward. Such a compromise was considered worthwhile to ensure confidence in the data interpretation.

## Conclusions

In summary, this study is the first to profile the systemic metabolic responses to increasing altitude in healthy humans, using untargeted metabolomics and lipidomics. Alterations identified in the aqueous fraction included the novel finding of decreasing isoleucine with ascent, with possible implications for muscle catabolism, alongside lactate and glucose changes that were in line with the well-documented increased reliance upon glycolytic energy metabolism. Fluctuations in the lipid profile with ascent suggest increased mobilisation of lipid stores and suppressed *de novo* lipogenesis. This occurred alongside changes to lipids that are essential membrane components, particularly those involved in the lipoprotein transport system. This study has therefore highlighted potential metabolic biomarkers for progressive hypoxic exposure in healthy humans.

### Ethics approval and consent to participate

The study design, risk management plan and individual protocols for the Caudwell Xtreme Everest (CXE) Expedition were approved by the University College London (UCL) Research Ethics Committee (in accordance with the declaration of Helsinki). Both verbal and written informed consent was obtained from all participants.

## Supplementary information

Supplementary Figures

## Data Availability

The datasets used and/or analysed during the current study are available from the corresponding authors on reasonable request.
