# Supplementary material for: Metabolomic and lipidomic plasma profile changes in human participants ascending to Everest Base Camp
Source: Sci Rep. 2019 Feb 19;9:2297. doi: 10.1038/s41598-019-38832-z (PMC6381113; doi:10.1038/s41598-019-38832-z)
Supplement: Supplementary file 1 — Supplementary Figures [file 41598_2019_38832_MOESM1_ESM.pdf]

Metabolomic and lipidomic plasma profile changes in human participants ascending to Everest Base Camp

O'Brien, K.A., Atkinson, R.A., Richardson, L., Koulman, A., Murray, A.J., Harridge, S.D.R., Martin, D.S, Levett, D.Z, Mitchell, K, Mythen, M.G, Montgomery, H.E, Grocott, M.P.W, Griffin, J.L., Edwards, L.M.

Supplementary Figures

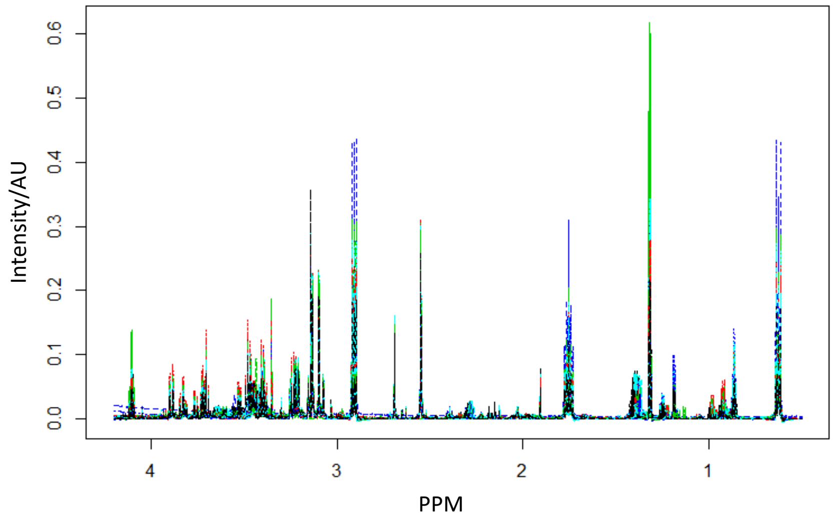

Supplementary figure 1. Aliphatic spectral region, overlaid.

An average of all spectra at all time points following alignment, PQ normalisation and scaling.

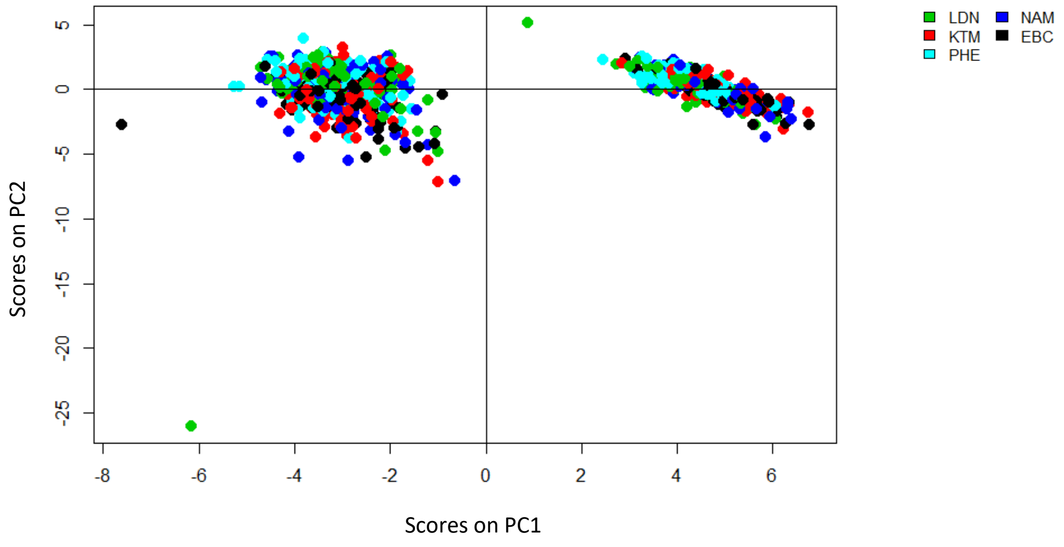

Supplementary figure 2. Principal component (PC) analysis scores plot.

Coloured according to altitude location, specificed in the figure key, defined as follows: LDN (London), KTM (Kathmandu), NAM (Namche), PHE (Pheriche), EBC (Everest Base Camp). PC analysis demonstrates the separation of groups based on a methodological artefact as oppose to experimental intervention. A clear batch effect is captured by PC1.

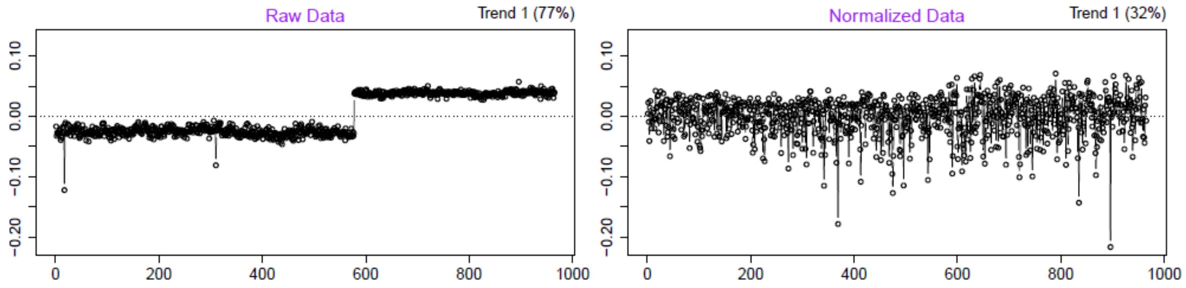

Supplementary figure 3. Identification of bias trend and correction using Eigen MS.

Bias trend 1 was found to capture 77% of the variance within the data (left panel), which was normalised using Eigen MS (right panel). Each dot is representative of a spectrum, plotted in time order. Units are arbitrary and correspond to the variance captured by the trend.

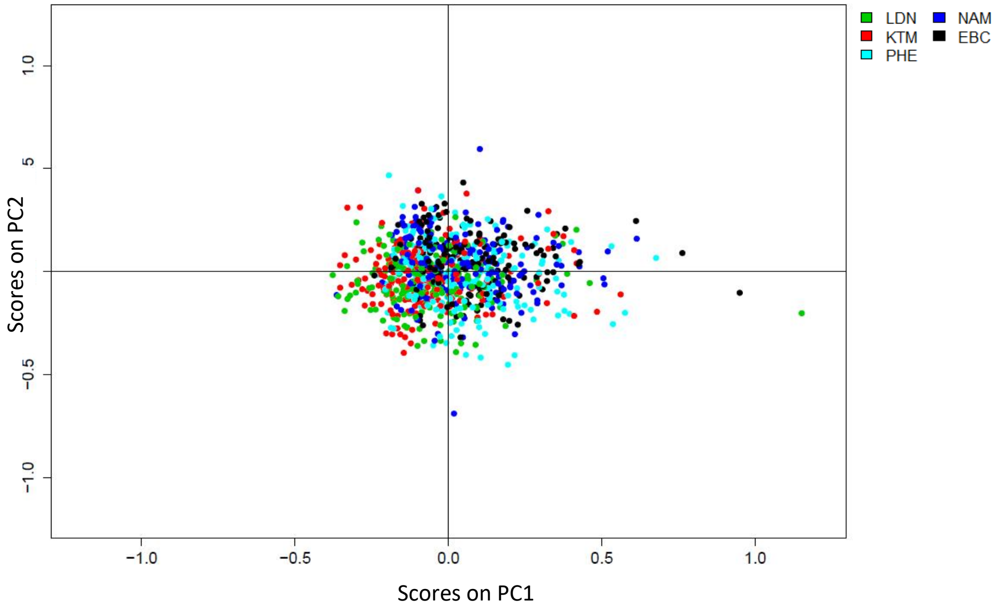

Supplementary figure 4. PCA scores plot following Eigen MS processing.

Coloured according to altitude location, specificed in the figure key, defined as follows: LDN (London), KTM (Kathmandu), NAM (Namche), PHE (Pheriche), EBC (Everest Base Camp).

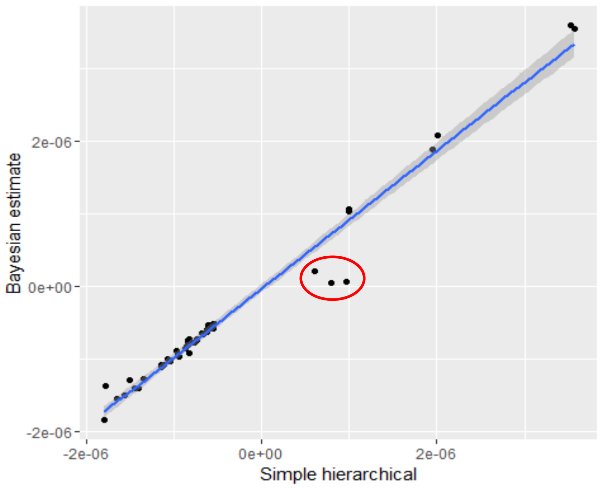

Supplementary figure 5. Slope (beta 1) estimates from a simple hierarchical model against a Bayesian estimation.

Highlighted in red are those slopes that have been overestimated by the rough method. The confidence interval around the regression is represented by the grey area surrounding the blue regression line.

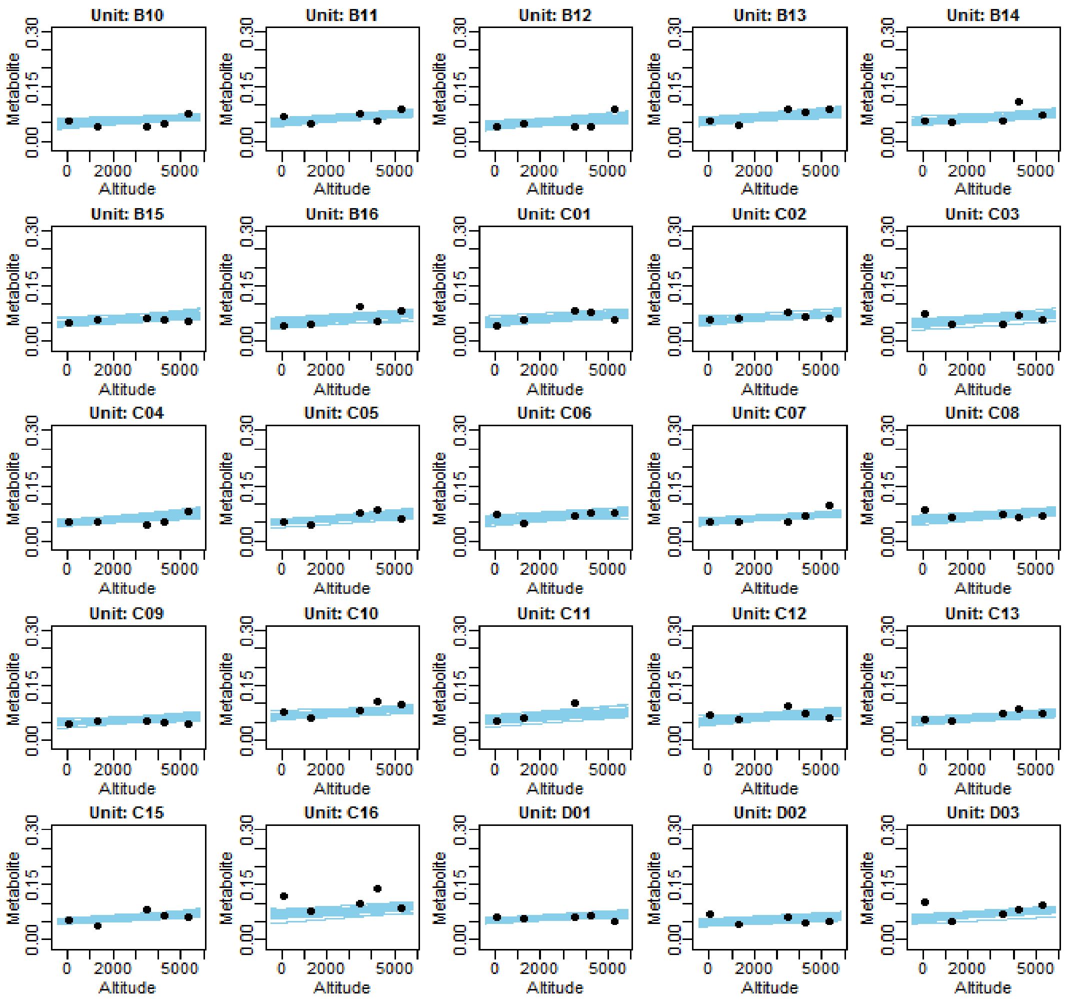

Supplementary figure 6. A select example of individual subject metabolite responses for lactate at each altitude.

Each individual is expressed as a 'unit' and the progressive change in metabolite abundance is expressed across each altitude. Representative regression lines in blue have been drawn from the fitted distribution. The most likely distribution from this individual response was then taken to inform the distribution of the group. The individual response is therefore constrained by the average response of the group

A.

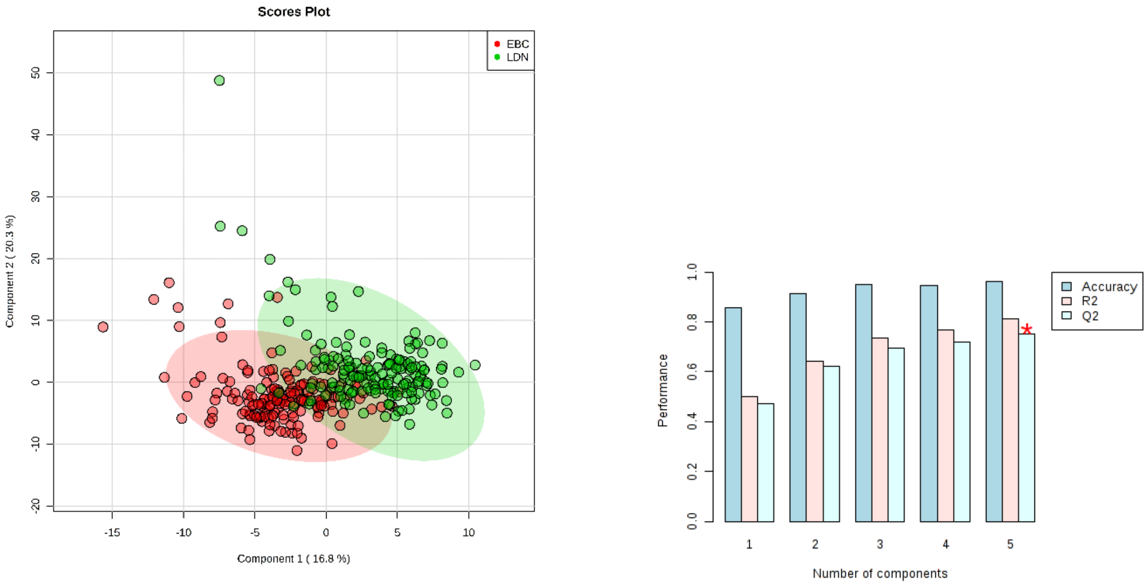
Scores Plot
EBC
LDN
Component 1 (16.8 %)
Component 2 (20.3 %)
Performance
Accuracy
R2
Q2
Number of components

B.

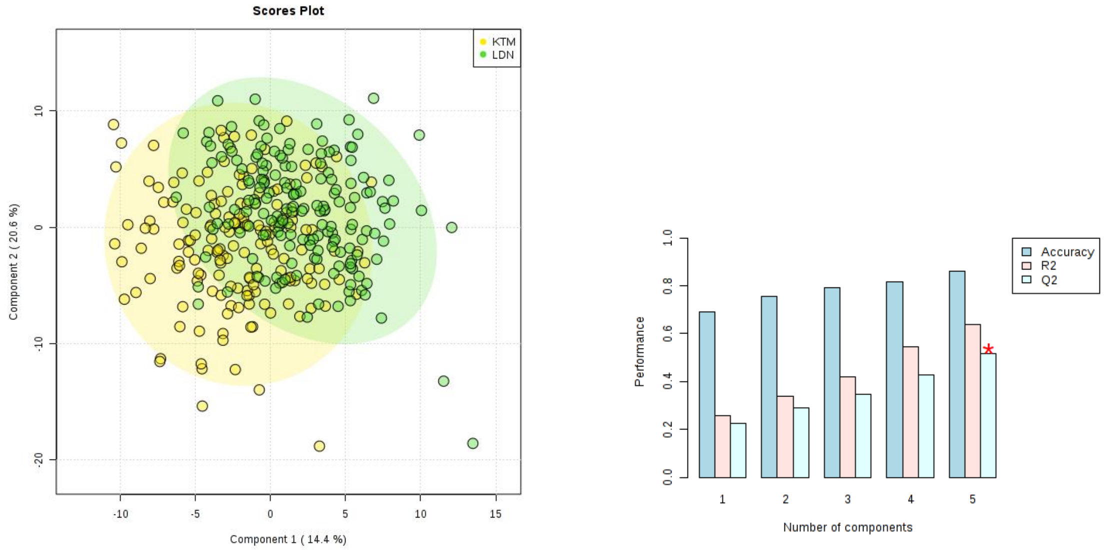
Scores Plot
KTM
LDN
Component 1 (14.4 %)
Component 2 (20.6 %)
Performance
Accuracy
R2
Q2
Number of components

C.

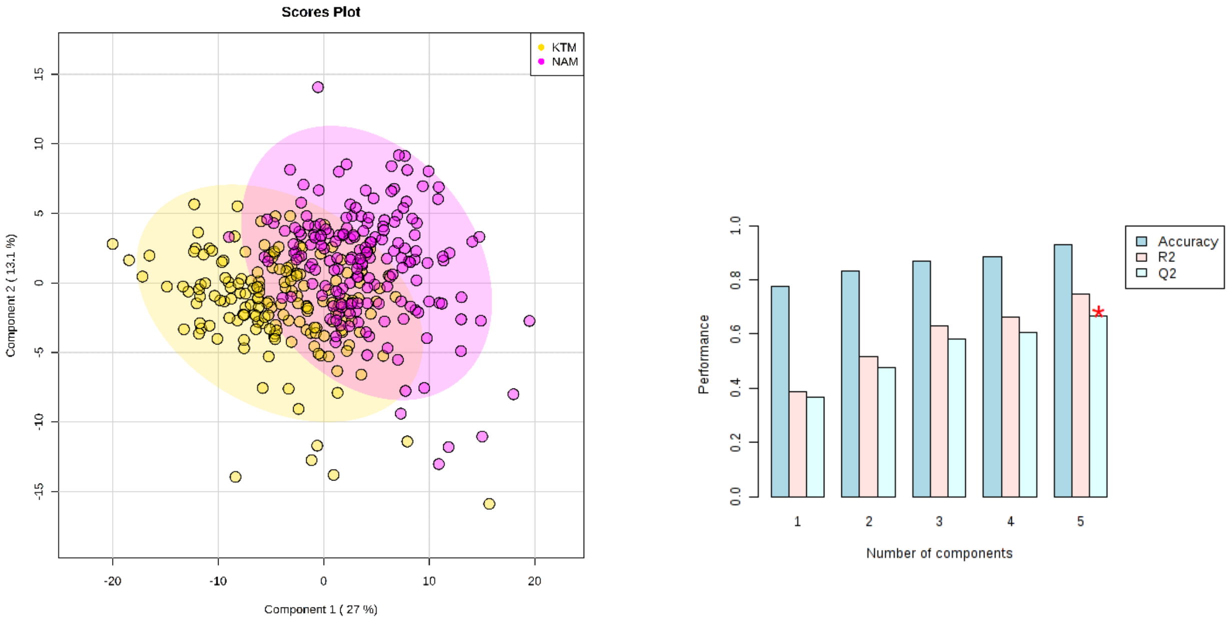

Supplementary Figure 7: PLS-discriminant analysis scores plots of lipid positive mode data presented alongside the corresponding cross validation model.

This initial analysis included comparison of the following altitude locations: London (LDN) and Everest Base Camp (EBC) ***(A)****, LDN and Kathmandu (KTM)* ***(B)****, KTM and Namche (NAM)* ***(C)****. Data were normalised using a combination of Pareto Scaling and Log Transformation. These plots were generated using Metaboanalyst: https://www.metaboanalyst.ca/MetaboAnalyst/faces/home.xhtml.*
